# Sensitivity of dose‐volume indices to computation settings in high‐dose‐rate prostate brachytherapy treatment plan evaluation

**DOI:** 10.1002/acm2.12563

**Published:** 2019-03-18

**Authors:** Marjolein C. van der Meer, Peter A. N. Bosman, Bradley R. Pieters, Yury Niatsetski, Niek van Wieringen, Tanja Alderliesten, Arjan Bel

**Affiliations:** ^1^ Department of Radiation Oncology Amsterdam UMC University of Amsterdam Amsterdam the Netherlands; ^2^ Life Sciences and Health Research Group Centrum Wiskunde & Informatica Amsterdam the Netherlands; ^3^ Algorithmics Group Department of Software Technology Faculty of Electrical Engineering, Mathematics and Computer Science Delft University of Technology Delft the Netherlands; ^4^ Physics and Advanced Development Elekta Veenendaal the Netherlands

**Keywords:** dose‐volume indices, HDR brachytherapy, prostate neoplasms, treatment planning

## Abstract

**Purpose:**

To investigate the variation in computed dose‐volume (DV) indices for high‐dose‐rate (HDR) prostate brachytherapy that can result from typical differences in computation settings in treatment planning systems (TPSs).

**Methods:**

Five factors were taken into account: number of dose‐calculation points, radioactive source description, interpolation between delineated contours, intersections between delineated organ contours, and organ shape at the top and bottom contour using either full or partial slice thickness. Using in‐house developed software, the DV indices of the treatment plans of 26 patients were calculated with different settings, and compared to a baseline setting that closely followed the default settings of the TPS used in our medical center. Studied organs were prostate and seminal vesicles, denoted as targets, and bladder, rectum, and urethra, denoted as organs at risk (OARs), which were delineated on MRI scans with a 3.3 mm slice thickness.

**Results:**

When sampling a fixed number of points in each organ, in order to achieve a width of the 95% confidence interval over all patients of the DV indices of 1% or less, only 32,000 points had to be sampled per target, but 256,000 points had to be sampled per OAR. For the remaining factors, DV indices changed up to 0.4% for rectum, 1.3% for urethra, and 2.6% for prostate. DV indices of the bladder changed especially if the high‐dose‐region was (partly) located at the most caudal contour, up to 8.5%, and DV indices of the vesicles changed especially if there were few delineated contours, up to 9.8%, both due to the use of full slice thickness for the top and bottom contour.

**Conclusions:**

The values of DV indices used in prostate HDR brachytherapy treatment planning are influenced by the computation settings in a TPS, especially at the most caudal part of the bladder, as well as in the seminal vesicles.

## 1 INTRODUCTION

High‐dose‐rate (HDR) brachytherapy is widely applied in the treatment of prostate cancer.^1^ An important tool in the evaluation and comparison of HDR prostate brachytherapy treatment plans are dose‐volume (DV) indices that describe the volumes of different organs receiving a certain dose. DV indices are an essential part of recent recommendations and guidelines for HDR prostate brachytherapy^1^, ^2^ as well as prospective treatment planning,^3^ making an understanding of the accuracy of such indices essential. Potential uncertainties in these indices should be taken into account when taking final decisions.

Many clinical and physical factors leading to uncertainties in the DV indices in brachytherapy in general have already been investigated.^4^ These include changes in geometry between treatment planning and delivery, the source strength calibration, and inter‐ and intra‐observer variability in image delineations of targets, organs at risk (OARs), and catheters. However, in addition to these uncertainties, settings in the algorithm for the computation of DV indices can also lead to differences.

A fundamental setting is the number of dose‐calculation points. The computation of DV indices is usually performed by calculating the dose in a number of points in the region of interest (ROI, either a whole organ or part of it) and assuming that these points are representative for the entire volume. These dose‐calculation points can for instance be placed in a regular grid spanning the ROI. However, the use of random sampling was argued to be superior for calculating DV indices.^5^ The number of points and their placement^6^ influences the values of the DV indices.

Another setting that can be varied is the source description, consisting of the dosimetric and geometrical data on the radioactive source. The dose in a point is typically calculated following the TG‐43 model, to which the source description is an input.^7^ The source description is based on previously done Monte Carlo simulations and measurements. It determines the dose in each dose‐calculation point and therefore the DV indices. The data in the source description is not exact, and improvements in simulations and measurements over time lead to updates in the source description such as mHDR‐v2,^8^ mHDR‐v2r,^9^ and mHDR‐v2c,^10^ revealing an inherent uncertainty in the source description.

A different type of setting is found in the representation of organs. The usual input of two‐dimensional contours does not uniquely define three‐dimensional organs. Therefore, the represented shape of the organ between two contours depends on the interpolation algorithm used. In the first reported DV histograms (DVHs) calculation method,^11^ the contours drawn on the two‐dimensional slices of the medical images were considered to fill the volume spanned by the scan, i.e., the delineated contour on the 2D slice was used for the entire slice thickness. Smoother organ surfaces can be obtained by using continuous interpolation.^12^

**Figure 1:**
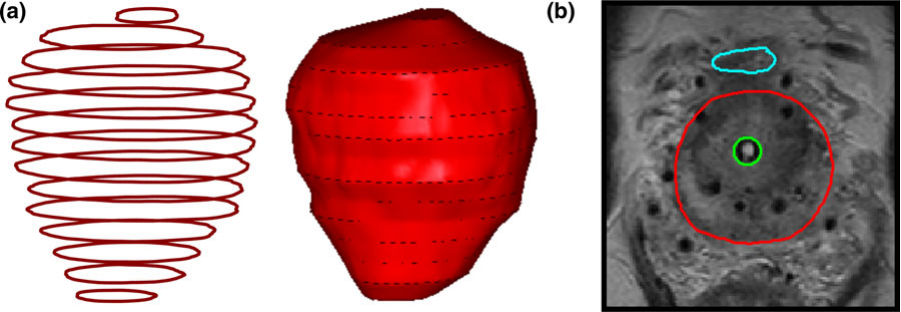
Illustration of interpolation algorithm and contour intersection. (a) The interpolation algorithm of the TPS used at our medical center, applied to delineated contours of a prostate (left) resulting in a three‐dimensional volume (right). (b) An axial slice from a magnetic resonance imaging (MRI) scan of a patient who underwent prostate brachytherapy with delineated prostate (red), bladder (turquoise), and urethra (green). The volume of the urethra can either be excluded from or included in the volume of the organ it intersects, namely the prostate.

Apart from the interpolation between contours, another setting in the organ representation is that the intersection between two organs can be considered to be part of both organs or of only one of them. Furthermore, the organ shape beyond the top and bottom contour can be defined by partial or full slice thickness. An example of a setting with continuous interpolation in combination with top and bottom contour cut‐off is shown in Fig. 1, together with an illustration of the setting for the intersection between two organs.

In general, variations in the settings for the computation of DV indices lead to different values and hence potentially to different decisions regarding treatment plans, i.e., to direct clinical impact. Moreover, a comparison of plans between different TPSs is difficult if the DV computation settings are different. The differences in DV indices between phantom‐based values and TPSs,^13^, ^14^ as well as between different commercial TPSs^15^ have been investigated before. However, the effects of different settings in the computation of DV indices on the DV‐index values of actual clinical treatment plans were not analyzed in such a manner. The aim of this study is to investigate the variation in computed DV indices that can result from typical differences in settings for the case of HDR prostate brachytherapy.

## 2 METHODS

There are two types of DV indices: volume and dose indices. Volume indices, i.e., the sub volume of an organ that receives at least (or at most) a specific dose, are useful for describing the volume of the tumor that receives a sufficiently high dose. Dose indices, i.e., the lowest dose to the most irradiated sub volume of a certain size of an organ, are useful for describing the amount of radiation delivered to OARs, as well as to targets. In this article, we use the following notation:

$V_{x\%}^{a}$: the volume of organ a that receives at least x% of the planning‐aim dose.

$D_{x\mathrm{cm}3}^{a}$: the lowest dose to the most irradiated x cm^3^ of organ a.

### 2.A Patient data

The patient data consisted of 26 consecutive patients who underwent prostate HDR brachytherapy at the medical center involved in this study between February 2015 and April 2017. The median age was 69.5 years with a range of 58–84. The median Gleason score was 7 (ISUP grade grouping 2–3) with a range of 6–10 (ISUP grade grouping 1–5). The urinary flow rate was reported for 23 patients, with a median of 17 ml/s and a range of 9.6–36.8 ml/s.

After catheter implantation, three orthogonal pelvic T2‐weighted turbo spin echo MRIs (Ingenia 3T Philips Healthcare, Best, the Netherlands) were acquired and used for treatment planning, with a resolution in the axial planes of 0.52 × 0.52 mm and a slice thickness of 3.3 mm (including a 0.3 mm gap). Only the axial slices were used for catheter reconstruction and delineation of ROIs; coronal and sagittal slices were used as an aid in the delineation. On most axial slices manual delineation was performed; on the remaining slices the delineations suggested by the interpolation algorithm of the clinical TPS were used. Only the base of the seminal vesicles was delineated.

The planning‐aim dose was $D_{90\%}^{\mathrm{prostate}}>13\mathrm{Gy}$. A median of 16 catheters was placed with a range of 14–20. The median number of activated dwell positions was 454.5 with a range of 250–668. The clinical criteria in terms of the values of DV indices employed in this study are shown in Table 1.

**Table 1:** DV indices and clinical criteria used for treatment planning. All patients involved in this study were treated at our medical center based on these criteria. Volume criteria V are relative to the total organ volume, dose criteria D are relative to the planning‐aim dose

Targets		OARs		
Prostate	Seminal vesicles	Bladder	Rectum	Urethra
$V_{100\%}>95\%$	$V_{80\%}>95\%$	$D_{1{\mathrm{cm}}^{3}}<86\%$	$D_{1{\mathrm{cm}}^{3}}<78\%$	$D_{0.1{\mathrm{cm}}^{3}}<110\%$
$D_{90\%}>100\%$		$D_{2{\mathrm{cm}}^{3}}<74\%$	$D_{2{\mathrm{cm}}^{3}}<74\%$	
$V_{150\%}<50\%$				
$V_{200\%}<20\%$				

### 2.B Clinical software

The TPS in which the clinically accepted plans for the patient group were created was Oncentra Brachy (version 4.3 or 4.5, Elekta AB., Stockholm, Sweden). In the TPS, DV indices were computed and evaluated in the “Brachy Planning” module.

### 2.C DV computation algorithm

Software for computing the DV indices of each patient used in this article was in‐house developed and validated with Oncentra Brachy. Validation was performed by calculating the dose in a fixed set of points in both systems. For one patient case, 5000 dose calculation points were equally distributed over the 5 ROIs (Table 1). Excluding points for which the distance to the active part of the source was less than 0.5 mm, the difference was below 0.08% of the prescribed dose.

The input of our in‐house developed software was the following information:

- Treatment date of the patient for determining the source strength.
- Delineated contours for the ROIs.
- Catheters information, including coordinates of the implanted catheters and source dwell positions and dwell times.
- Source information, including TG‐43 data describing the source.

In the following sections we describe components of the software that play a key role in computing DV indices.

#### 2.C.1 Dose‐calculation points

The placement of dose‐calculation points was done by uniform random sampling inside an ROI. To this end, for each ROI, a bounding box was created which completely enclosed the ROI. Next, points were sampled in this box uniformly randomly and only points which were within the ROI were accepted, i.e., rejection sampling was used. Sampling was continued until the desired number of points inside the ROI (allowing points to be on the surface of the ROI) was reached.

In the TPS used in our medical center, random sampling is performed with a fixed seed for the random number generator, essentially making the algorithm deterministic. Moreover, a fixed number of sample points per ROI is used. This approach introduces a dependency of the precision of a DV index on the volume it pertains to, both for volume indices and for dose indices, in the following way.

For volume indices, when a total of n points is sampled in an ROI, of which a fraction p consists of the volume corresponding to the DV index, then the number of points inside the volume of the DV index follows a binomial distribution. The probability that k of the n sampling points will be inside the volume of the DV index is equal to $P\left(X=k\right)=\left(\begin{array}{c} n\\ k \end{array}\right)p^{k}{\left(1-p\right)}^{n-k}$ with an average of μ=np and a variance of $\sigma^{2}=np\left(1-p\right).$ The closer p is to 0.5, the larger the variance. Hence, the variance of the DV indices is based on how close the volume of the DV index is to 50% of the organ volume.

For dose indices, when sampling a number of points per ROI, the variance depends on the number of sample points that are in the volume of the DV index. This means that the variance of these DV indices is based on the number of sample points per cm^3^.

We considered the impact of the number of sample points used for the dose calculation on the precision of the DV indices. Following the approach of the TPS used in our medical center, a fixed number of sample points per ROI was used. However, in order to eliminate the dependency of the precision of dose indices on the relevant volumes, we additionally considered using a fixed number of sample points per cm^3^ for dose indices.

#### 2.C.2 Radioactive source description

Dose calculation was based on the update of the AAPM Task Group No.43 dose formalism.^16^ The radial dose function and anisotropy function were based on previously done Monte Carlo simulations of the 192‐Iridium source. In clinical treatment planning, the mHDR‐v2 source description^8^ was used. Because of the small design change made by the manufacturer after this first study, resulting in a small change to the source used in clinical practice, new dosimetric data has been provided,^9^ resulting in the mHDR‐v2r source description. Both studies were then taken into account in the publishing of a consensus file, the mHDR‐v2c source description.^10^ We considered the impact of using each of these three different source description files.

#### 2.C.3 Contour interpolation

A straightforward way of defining a three‐dimensional volume from two‐dimensional contours made on individual slices is to assume that each contour fills the volume in the z‐direction spanned by the slice (i.e., slice thickness). This approach assumes that MRI (or computed tomography, CT) scan slices represent usually an average over the slice thickness. To obtain smoother organ surfaces, an interpolation algorithm can be used. In order to study the influence of interpolation, we applied shape‐based interpolation using a chamfer distance,^12^ which is the interpolation method implemented in our clinically used TPS (Fig. 1).

The algorithm used for interpolation between contours of an ROI used a volume grid.^12^ For the interpolation between two contours at height z_1_ and z_2_, a two‐dimensional grid was placed on each of the contours. For each point in a slice, the smallest Euclidean distance to the contour in that slice was calculated, where the distance is positive if the point is inside the contour and negative otherwise. Next, linear interpolation was performed between each pair of corresponding grid points on the two contours to obtain the value of that grid point at height z = (z_1_ + z_2_)/2. Finally, we used the marching squares algorithm^17^ to obtain the contour at height z.

For all patients involved in this study, the grid spacing in the clinically used TPS was set to “auto spacing”, giving a spacing of 0.82 mm. The same spacing was used in our software. The interpolation algorithm was used for all pairs of consecutive contours on the MRI slices. This way, an interpolated contour was added half‐way between each pair of delineated contours. After this, each contour was assumed to fill half the volume that the slice spanned.

#### 2.C.4 Including or excluding contour intersection

The intersection between two contours can be assumed to be either a part of both ROIs or only a part of one of the ROIs. For prostate brachytherapy in particular, intersections exist between the prostate and the urethra, as well as between the bladder and the urethra. The urethra can thus either be considered to be a part of both the prostate and the bladder or none of them. The clinically used TPS supports both possibilities. Being part of both organs is the default option.

Since the urethra passes through the prostate, there is always overlap between the delineated contours of the urethra and the prostate. Moreover, for our patient data, there was overlap between the delineated contours of the urethra and the bladder as well. The reason for this is that the urethra was delineated as the part of the urinary catheter through the prostate into the bladder, due to the fact that the urethra itself is often not well visible on the MRI. Because our clinical TPS by default considers the urethra as a part of the organs it intersects, the delineations of the urethra inside the bladder were redundant, but gave an overlap between bladder and urethra. All delineations of the urethra, including those inside the bladder, were taken into account in this study.

#### 2.C.5 Partial or full top and bottom slice thickness

**Figure 2:**
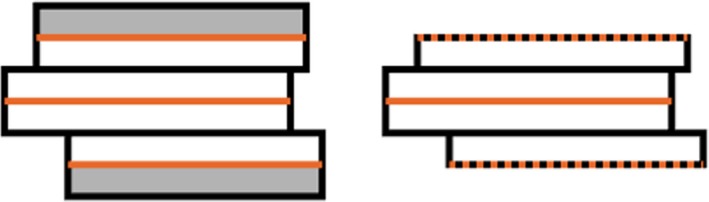
Illustration of slice thickness. Full top and bottom slice thickness (left) versus partial slice thickness (right) for an ROI with three delineated contours (in orange). The difference in resulting volume consists of the gray parts in the left shape.

The organ shape at the top and bottom contour can be defined by considering the top and bottom slice to fill the volume in the z‐direction spanned by the slice. In this case, there is full slice thickness at the most cranially and the most caudally located contour of the organ. Conversely, Oncentra Brachy^1^ assumes the top and bottom contour to be part of the surface of the organ: i.e., the ROI does not extend beyond the top and bottom contour, and partial slice thickness is used. The difference between the two settings is relatively large for an organ that consists of only a few contours, as shown in Fig. 2.

### 2.D Volume and dose indices

For the computation of DV indices, the clinically used TPS employs binning of the DVH. However, in our in‐house developed software, we used an approach where binning is unnecessary.^18^ For a volume index, the number of dose‐calculation points where the dose is larger or smaller than a specific dose was counted. Dividing this number by the total number of dose‐calculation points in that organ gave the relative volume of an organ receiving at least or at most that dose. For a dose index, the dose‐calculation points were sorted from highest to lowest dose. The dose value of the first point in this sorting that corresponded to the required volume, was returned. For example, if 1,000 dose‐calculation points are sampled in a urethra of 2.01 cm^3^, then the $D_{0.1\mathrm{cm}3}^{\mathrm{urethra}}$ is determined by the dose of point 50 in the sorted list.

### 2.E Analysis

When computing DV indices, we studied the influence of five factors.

1. The number of dose‐calculation points per ROI used in random sampling.
2. Dosimetric data for mHDR‐v2, mHDR‐v2r, or mHDR‐v2c source models.
3. Whether interpolation was used between pairs of consecutive delineated contours.
4. Whether the urethra was considered to be part of the intersecting organs.
5. Full or partial slice thickness inclusion at the top and bottom contour.

For a given number of dose‐calculation points, we defined a baseline setting that closely followed the default settings in the clinical TPS. Specifically, in the baseline setting, the mHDR‐v2 dosimetric data was used, the urethra was considered part of the prostate and bladder, contour interpolation was used, and partial slice thickness was used at the top and bottom contour.

Because the number of dose‐calculation points was not a categorical variable, we first studied this factor separately, using the baseline settings for the other factors. By considering the number of points that were actually located inside an organ, the result was independent of the bounding box that was used for sampling points in that organ.

For a given number of dose‐calculation points, the DV indices were computed 100 times using a pseudo‐random number generator with different random seeds (the Mersenne Twister 19937^19^). The variance was used to calculate the width of a 95% confidence interval (CI). Since the sampled points follow a binomial distribution which rapidly converges to a normal distribution for many dose‐calculation points, a normal distribution was assumed. The result was averaged over all patients.

The DV indices were computed for 1,000–256,000 dose‐calculation points for both targets and OARs, each step doubling the number of dose‐calculation points. The DV indices of the OARs were additionally computed with a fixed number of dose‐calculation points per cm^3^, where the total number of dose‐calculation points in these organs depended on the delineated volume of each of the ROIs. The DV indices were computed for 10–2,560 dose‐calculation points per cm^3^, each step doubling the number of dose‐calculation points. For each number of dose‐calculation points, the result was averaged over all patients.

By using a large number of dose‐calculation points, the true influence of the remaining factors could be studied. We fixed the number of dose‐calculation points to 256,000 per target, and the number of dose‐calculation points in OARs to 2,560 dose‐calculation points per cm^3^. First, we studied only the impact of changing the dosimetric data from mHDR‐v2 to either mHDR‐v2r or mHDR‐v2c. Then, we studied the impact of the remaining three factors, which resulted in a total of eight possible settings. All results were compared to the baseline setting. For the most influential factors, the influence compared to the baseline setting was tested using a paired statistical test, selected based on the data. The significance threshold was set at 0.01. Normality of the data of two variables was tested using a Q‐Q plot; symmetry of the data was tested using a boxplot of the difference between two variables.

## 3 RESULTS

**Figure 3:**
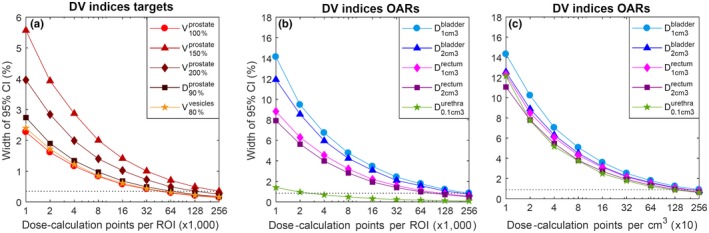
The uncertainty in the DV indices for targets and OARs as a function of the number of dose‐calculation points. The width of the 95% confidence interval (CI) is either in percentage of total ROI volume for volume indices V, or percentage of planning‐aim dose ($D_{90\%}^{\mathrm{prostate}}>13\text{Gy}$) for dose indices D. On top the result for the targets (prostate and seminal vesicles) is shown. On the bottom the result for the OARs is shown. The dotted lines indicate the upper bound on the width of the 95% CI for the highest number of dose‐calculation points.

The width of the 95% CI of each DV index as a function of the number of dose‐calculation points when considering the baseline setting is shown in Fig. 3. When the number of dose‐calculation points is fixed per target, the ordering of the confidence interval of volume indices of targets from large to small is $V_{150\%}^{\mathrm{prostate}}$, $V_{200\%}^{\mathrm{prostate}}$, $V_{80\%}^{\mathrm{vesicles}}$, $V_{100\%}^{\mathrm{prostate}}$, i.e., ordered on how close on average the volume of the DV index is to 50% of the organ volume. When the number of dose‐calculation points is fixed per cm^3^, the total number per OAR depends on the OAR volume. The average delineated volume of each of the ROIs in the baseline setting is shown in Table 2.

**Table 2:** The volume of targets and OARs over all patients, determined in the baseline setting

ROI	Average (cm^3^)	Minimum (cm^3^)	Maximum (cm^3^)
Prostate	33.97	16.46	71.09
Seminal vesicles	4.08	0.51	11.79
Bladder	98.48	44.50	264.64
Rectum	54.04	23.16	108.54
Urethra	2.08	0.51	3.81

The differences over all patients for the setting of the dosimetric data of the source with respect to the baseline setting for DV indices of prostate, seminal vesicles, and OARS, are very small. The maximum difference was observed for the seminal vesicles with 0.94% (Supplementary material, Fig. S1).

**Figure 4:**
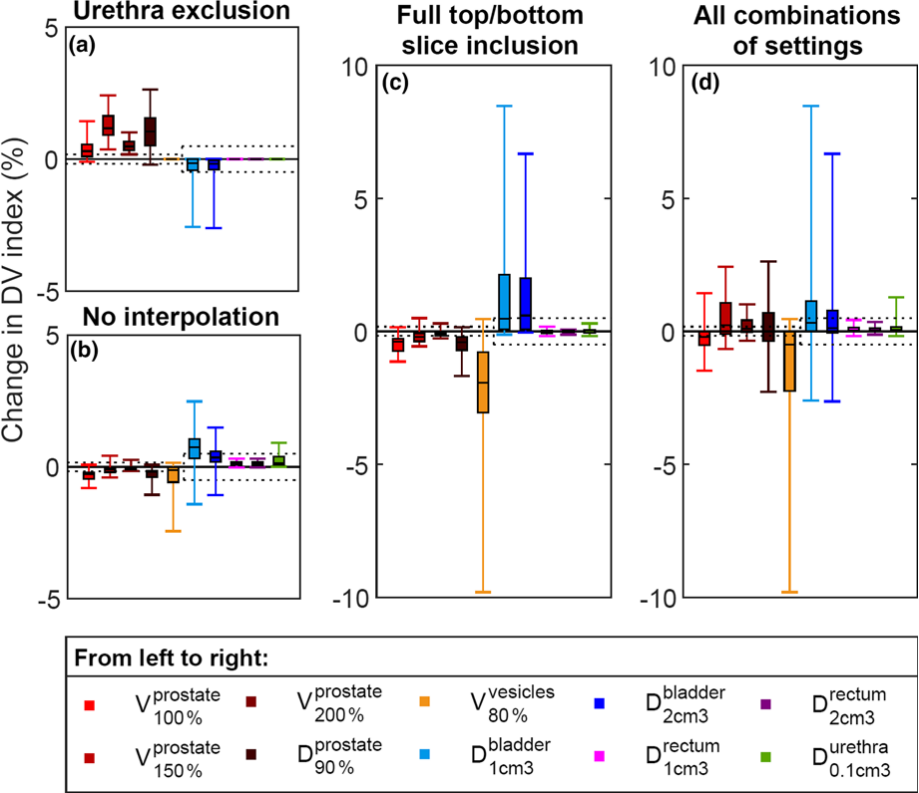
The sensitivity of DV indices for both targets and OARs to different ROI representation settings. Each result is relative to the baseline setting. The change in DV index is presented as percentage of total ROI volume for volume indices V, or percentage of planning‐aim dose ($D_{90\%}^{\mathrm{prostate}}>13\mathrm{Gy}$) for dose indices D. Dotted lines show the 95% CI associated with the uncertainty related to the sampling of dose‐calculation points. Each boxplot shows the distribution over all patients (median at 50%, box from 25% to 75%, whiskers at 0% and 100%).

The differences in the other settings are shown in Fig. 4. Because the urethra was delineated as the urinary catheter, there could be overlap between the ROI delineated as the urethra, and the bladder. The most sensitive DV indices were found to be the $V_{80\%}^{\mathrm{vesicles}}$, and the $D_{1{\mathrm{cm}}^{3}}^{\mathrm{bladder}}$ and $D_{2{\mathrm{cm}}^{3}}^{b\mathrm{ladder}}$ for the bladder. Multiple settings resulted in a $V_{80\%}^{\mathrm{vesicles}}$ which was lower than in the baseline setting. Vice versa, multiple settings resulted in a $D_{1{\mathrm{cm}}^{3}}^{\mathrm{bladder}}$ and $D_{2{\mathrm{cm}}^{3}}^{\mathrm{bladder}}$ which were higher than in the baseline setting. For all three DV indices, the treatment plan thus appeared to be worse in other settings than in the baseline setting. In extreme cases, as shown in Fig. 4(c), the difference could become an increase of 6.7% in the $D_{2{\mathrm{cm}}^{3}}^{\mathrm{bladder}}$, an increase of 8.5% in the $D_{1{\mathrm{cm}}^{3}}^{\mathrm{bladder}}$, and a decrease of 9.8% in the $V_{80\%}^{\mathrm{vesicles}}$ with respect to the baseline setting.

Moreover, for the prostate, the $V_{150\%}^{\mathrm{prostate}}$ and $D_{90\%}^{\mathrm{prostate}}$ were also relatively sensitive as shown in Fig. 4(d). The sensitivity of the DV indices of rectum and urethra was within the variance of the number of dose‐calculation points.

For the prostate, the inclusion or exclusion of the urethra was the most important factor [Fig. 4(a)]. On average, the amount of radiation to the urethra was between 100% and 110% of the planning‐aim dose. The relative volume of the urethra that received 100% of the planning‐aim dose was close to the relative volume of the prostate (excluding the urethra) that received 100% of the planning‐aim dose, so the $V_{100\%}^{\mathrm{prostate}}$ remained similar after exclusion of the urethra. However, an important factor in making the treatment plans was urethra sparing, i.e., minimizing the dose to the urethra. Therefore, the dose in the urethra was on average lower than the dose in the prostate (excluding the urethra). Hence, excluding the urethra increased the $D_{90\%}^{\mathrm{prostate}}$.

For the $V_{150\%}^{\mathrm{prostate}}$ and the $V_{200\%}^{\mathrm{prostate}}$, the *relative* volume receiving over 150% or 200% of the planning‐aim dose is used. However, the *absolute* volume receiving over 150% or 200% of the planning‐aim dose was in practice independent of whether the urethra was included in the prostate. Since the DV index was calculated with respect to the total volume, the larger this total volume, the larger the difference in DV index between including and excluding the urethra. Since the volume receiving over 150% of the planning‐aim dose includes the volume receiving over 200% of the planning‐aim dose, the $V_{150\%}^{\mathrm{prostate}}$ was more sensitive to urethra exclusion than the $V_{200\%}^{\mathrm{prostate}}$.

For the two most influential factors, the difference compared to the baseline setting was tested using a paired statistical test based on the data. The data of the $V_{150\%}^{\mathrm{prostate}}$, $D_{90\%}^{\mathrm{prostate}}$, $D_{1\mathrm{cm}3}^{\mathrm{bladder}}$ and $D_{2\mathrm{cm}3}^{\mathrm{bladder}}$ were normally distributed, hence a paired samples t‐test was used. The 95% CI of the difference between inclusion and exclusion of the urethra in the prostate was (1.1, 1.5) for the $V_{150\%}^{\mathrm{prostate}}$ (*P *<* *0.001) and (0.8, 1.4) for the $D_{90\%}^{\mathrm{prostate}}$ (*P *<* *0.001). Concerning the difference between full and partial slice thickness, for the bladder it was (0.6, 2.4) for the $D_{1\mathrm{cm}3}^{\mathrm{bladder}}$ (*P *=* *0.002), and (0.6, 2.1) for the $D_{2\mathrm{cm}3}^{\mathrm{bladder}}$ (*P *=* *0.001). The data of the $V_{80\%}^{\mathrm{vesicles}}$ was not normally distributed, nor was the difference symmetrical in shape, hence a paired‐samples sign test was used. For the $V_{80\%}^{\mathrm{vesicles}}$, there was a statistically significant median decrease using full slice thickness (−2.6%) compared to partial slice thickness (*P *<* *0.001).

## 4 DISCUSSION

In this study, the influence of computation settings on the resulting DV indices of clinically optimized HDR prostate brachytherapy plans was investigated. These settings were related to number of dose‐calculation points, dosimetric data (source models), and organ representation, and can differ between TPSs^2^^,^ 15. Differences in DV indices of up to 9.8% were observed.

### 4.A Dose‐calculation points

The study showed that a large number of dose‐calculation points is required for the DV indices of the OARs to be accurate (i.e., have little uncertainty). When sampling a fixed number of points in an organ, in order to achieve a width of the 95% CI of 1% or less, only 32,000 points have to be sampled per target, but 256,000 points have to be sampled per OAR. This is due to the use of dose indices of an absolute volume which is small compared to the total volume of the OAR, such as the $D_{1\mathrm{cm}3}^{\mathrm{bladder}}$.

The large number of dose‐calculation points that is required for the DV indices of OARs to reach high accuracy is in accordance with previous studies,^6^ where it was recommended not to use the $D_{\min}$ and the $D_{\max}$ to describe dose distributions because of their large sensitivity to the number of dose‐calculation points. More dose‐calculation points result in a more accurate result, but also a slower calculation. The uncertainty of grid sampling versus random sampling has been studied before and is in general even higher.^5^

### 4.B Radioactive source description

The maximum difference resulting from different dosimetric data of the source^8^, ^9^, ^10^ was observed for the seminal vesicles with 0.94%, making this uncertainty in the range of the uncertainty of the dose‐calculation points. A newer version of the dosimetric data can be assumed to be better, but the influence of this setting is negligible.

### 4.C Organ intersections

The setting in our study that influenced the DV indices of the prostate the most was whether or not to include the urethra in the prostate. Excluding the urethra from the prostate increased the DV indices of the prostate, especially the $D_{90\%}^{\mathrm{prostate}}$ and the $V_{150\%}^{\mathrm{prostate}}$ with medians of 1.0%, respectively, 1.2%.

This setting differs between TPSs. Oncentra Brachy by default considers the urethra to be part of the prostate. In contrast, Oncentra Prostate excludes the urethra from the prostate, as well as Vitesse, a TPS specifically for HDR prostate (Varian Medical Systems, Charlottesville, VA, USA).

This setting should be carefully considered by a medical center, before designing treatment plans based on a certain clinical protocol. Excluding the urethra from the prostate not only influences the DV index values, it also affects the clinical dose aims used during treatment planning that apply to the urethra. If the urethra is included in the prostate, then the requirement $V_{100\%}^{\mathrm{prostate}}>95\%$ comprises the dose to the urethra as well. However, if the urethra is excluded from the prostate, then the only aim would be $D_{0.1\mathrm{cm}3}^{\mathrm{urethra}}<110\%$ of the planning‐aim dose. Due to uncertainties, it could then be prudent to define a lower limit on the dose, e.g., on $D_{0.1\mathrm{cm}3}^{\mathrm{urethra}}$.

### 4.D Contour interpolation and slice thickness inclusion at the outer contours

For the settings of both contour interpolation and slice thickness at the outer contours, especially the DV indices of the seminal vesicles and the bladder were sensitive. Without the use of contour interpolation, the median difference in the DV indices was at most 0.75%, which was observed for the $D_{1\mathrm{cm}3}^{\mathrm{bladder}}$. Extremes were at most 2.5%, observed for both the $V_{80\%}^{\mathrm{vesicles}}$ and the $D_{1\mathrm{cm}3}^{\mathrm{bladder}}$. Using full top and bottom slice thickness, a median decrease of 2.6% was seen for the $V_{80\%}^{\mathrm{vesicles}}$. Extremes include an increase of 8.5% for the $D_{1\mathrm{cm}3}^{\mathrm{bladder}}$, and a decrease of 9.8% for the $V_{80\%}^{\mathrm{vesicles}}$.

For the seminal vesicles, the sensitivity of the DV indices to these settings could be explained by the small target volume, in combination with the large surface of the top and bottom contour. This is also because usually only the base of the vesicles is delineated. For the bladder, the most irradiated 1 cm^3^ and 2 cm^3^ were often exactly at the bottom contour. The importance of the 3D reconstruction algorithm at the outer slices has been noted before in a phantom study.^15^

Still, settings differ between different TPSs. Oncentra Brachy uses interpolation in combination with partial slice thickness. Vitesse uses a different interpolation algorithm, also in combination with partial slice thickness. Other TPSs of Varian use again different settings. VariSeed uses full slice thickness, whereas BrachyVision has been reported to round the ends of cylinder edges contained within the last slice^15^ because of the use of interpolation where ROI boundaries are smoothed.

This uncertainty can be improved by using a smaller slice thickness, that can be obtained without loss of anatomic detail,^20^ and delineating more contours. In addition, the evaluation of a treatment plan is often a combination of evaluating the DV indices and visually inspecting the projections of the 3D dose distribution on the MRI scans. Especially if the high‐dose region of the bladder is (partly) located at the bottom contour, and/or the seminal vesicles have few delineated contours, their DV indices may be unreliable to evaluate a treatment plan. The visual inspection of the 3D dose distribution especially at the boundary area of the bladder, as well as in the vesicles, is then needed for a reliable evaluation.

### 4.E Clinical impact

A limitation of this study is that it is a single‐center study. The described treatment plans were optimized in our clinical TPS (i.e., Oncentra Brachy) with its default settings and could give different results if the treatment plans had been optimized in other TPSs. However, our finding that computation settings can influence the DV index values is general. The clinical relevance of this uncertainty in the DV index values depends on the total planned dose. If patients receive brachytherapy next to external beam radiotherapy, the deviations are a smaller part of the total dose than if brachytherapy is given as monotherapy. The study is limited to prostate brachytherapy, but similar results may occur even for different treatment sites.

This study has been performed retrospectively. However, computation settings may influence the optimization process inherent in treatment planning, be it manual or automated.^21^, ^22^ It would be interesting to also consider the magnitude of this influence on the optimization process and the outcome thereof. Moreover, treatment plan optimization could possibly be adapted to account for this influence by applying robust optimization to these uncertainties. Treatment plan optimization whereby the influence of different computation settings is accounted for, is suggested as future work.

## 5 CONCLUSIONS

The values of DV indices used in prostate HDR brachytherapy treatment planning are influenced by the computation settings in a TPS, especially at the most caudal part of the bladder, as well as in the seminal vesicles, potentially to an extent that it could influence decisions on final treatment plan construction.

## CONFLICT OF INTEREST

This work was partly funded by Elekta AB (Stockholm, Sweden). Dr. Alderliesten, Dr. Bel, Prof. Dr. Bosman, and Dr. Pieters are leaders of projects supported by Elekta. Y. Niatsetski is an Elekta employee.

## Supporting information

Fig. S1. The sensitivity of DV indices for both targets and OARs to different source models, relative to the baseline setting (mHDR‐v2^8^). The change in DV index is presented as percentage of total ROI volume for volume indices *V*, or percentage of planning‐aim dose ($D_{90\%}^{\mathrm{prostate}}>13Gy$) for dose indices *D*. Dotted lines show the 95% confidence interval associated with the uncertainty related to the sampling of dose‐calculation points. Each boxplot shows the distribution 10 over all patients (median at 50%, box from 25% to 75%, whiskers at 0% and 100%).

## NOTES

1 Nucletron B.V., *Oncentra Brachy v4.5 Physics and Algorithms*.

2 Nucletron B.V., *Oncentra Brachy v4.5 Physics and Algorithms*. Nucletron B.V., *Oncentra Prostate v4.2 Reference Manual*. Varian Medical Systems, Inc., *Vitesse v4.5 Reference Guide*. Varian Medical Systems, Inc., *Brachy Vision 15.5 Algorithms Reference Guide*.
