# Supplementary material for: Sensitivity of dose‐volume indices to computation settings in high‐dose‐rate prostate brachytherapy treatment plan evaluation
Source: J Appl Clin Med Phys. 2019 Mar 18;20(4):66–74. doi: 10.1002/acm2.12563 (PMC6448172; doi:10.1002/acm2.12563)
Supplement: Supplementary file 1 — Fig. S1. The sensitivity of DV indices for both targets and OARs to different source models, relative to the baseline setting (mHDR‐v28). The change in DV index is presented as percentage of total ROI volume for volume indices V, or percentage of planning‐aim dose (D90%prostate>13Gy) for dose indices D. Dotted lines show the 95% confidence interval associated with the uncertainty related to the sampling of dose‐calculation points. Each boxplot shows the distribution 10 over all patients (median at 50%, box from 25% to 75%, whiskers at 0% and 100%). [file ACM2-20-66-s001.pdf]

Sensitivity of dose-volume indices to computation settings in HDR prostate brachytherapy treatment plan evaluation

Supplementary Material

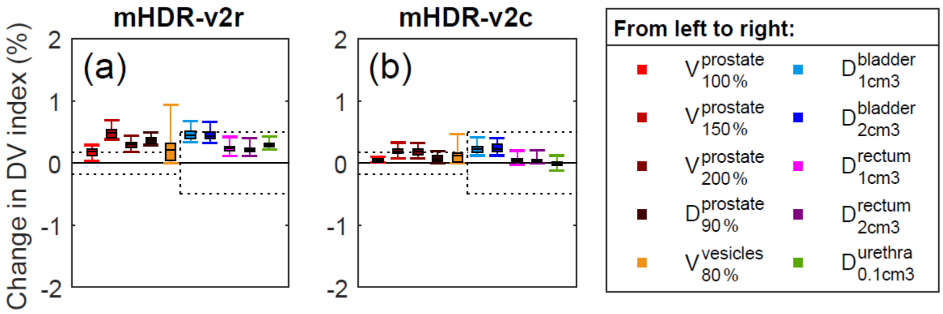

Fig.1. The sensitivity of DV indices for both targets and OARs to different source models, relative to the baseline setting (mHDR-v2[8]). The change in DV index is presented as percentage of total ROI volume for volume indices V, or percentage of planning-aim dose ($D_{90\%}^{prostate} \geq 13$ Gy) for dose indices D. Dotted lines show the 95% confidence interval associated with the uncertainty related to the sampling of dose-calculation points. Each boxplot shows the distribution over all patients (median at 50%, box from 25% to 75%, whiskers at 0% and 100%).
